# Association between household secondhand smoke exposure and ADHD in US children aged 4–15 years: Evidence from NHANES 1999–2004

**DOI:** 10.18332/tid/222368

**Published:** 2026-07-16

**Authors:** Yongzheng Bao, Wanchao Zhang, Chunhui Wang, Xia Zhang, Xiaoju Hou, Zhibang Hu

**Affiliations:** 1 Department of Otorhinolaryngology, Changzhou Third People's Hospital, Changzhou, China; 2 Changzhou Clinical College, Xuzhou Medical University, Changzhou, China; 3 Department of Radiology, The People’s Hospital of Wuqia, Kezhou, China; 4 Department of Eye and ENT, The People’s Hospital of Wuqia, Kezhou, China

**Keywords:** household smoke exposure, ADHD, children, NHANES, epidemiology

## Abstract

**INTRODUCTION:**

Household tobacco smoke (or secondhand smoke, SHS) exposure is a common type of environmental exposure among children, and it may be associated with the development of attention deficit hyperactivity disorder (ADHD); however, current findings remain inconsistent. This study aimed to examine the association between household SHS exposure and ADHD among US children, and to provide an epidemiological foundation for ADHD prevention and tobacco-control interventions targeting pediatric populations.

**METHODS:**

This is a secondary analysis of pooled secondary data from the National Health and Nutrition Examination Survey (NHANES) 1999–2004. ADHD cases were identified through questionnaire-based assessments, and participants were categorized into exposed and non-exposed groups according to whether or not any household member smoked inside the home. Weighted multivariate logistic regression models were employed to evaluate the association between household SHS exposure and ADHD. In addition, subgroup and sensitivity analyses were conducted.

**RESULTS:**

A total of 6790 children were included in the analysis, of whom 452 (7.97%) were classified as having ADHD. The prevalence of household SHS exposure was significantly higher among children with ADHD compared to those without (36.25% vs 22.77%, p<0.001). After adjustment for multiple confounding variables, household SHS exposure remained significantly associated with ADHD (OR=1.583; 95% CI: 1.166–2.150). Subgroup analyses stratified by sex, age, race, poverty–income ratio (PIR), body mass index (BMI), and birth weight showed that the association remained generally consistent across all strata (all p for interaction >0.05). Sensitivity analyses further confirmed the positive association between household SHS exposure and ADHD (all p<0.05).

**CONCLUSIONS:**

Household SHS exposure was significantly associated with ADHD among US children within our cross-sectional analysis. Future prospective investigations are required to clarify the causal relationship underlying this association.

## INTRODUCTION

Attention deficit hyperactivity disorder (ADHD) is among the most prevalent neurodevelopmental disorders found in children and is characterized by inattention, hyperactivity, and impulsive behavior^1^. The global prevalence of ADHD among children and adolescents has been estimated to be approximately 8%, and it continues to increase^2-4^. ADHD symptoms frequently persist into adulthood, contributing to academic underachievement, impaired interpersonal relationships, and an elevated risk of accidental injury^5^. Consequently, ADHD imposes substantial economic and psychological burdens on both families and society. Although the etiology of ADHD remains incompletely understood, the disorder is generally considered to arise from complex interactions between genetic susceptibility and environmental exposures^6^. Among numerous environmental toxicants, SHS has attracted considerable attention because of its widespread prevalence and the well-established neurotoxic effects of its constituents on the developing brain. Approximately 23.3% of children in the United States (more than 11 million) were estimated to have been exposed to secondhand smoke between 2021 and 2023^7^. Accordingly, SHS exposure has become an important focus of epidemiological and clinical research^8,9^.

SHS contains thousands of chemical compounds, including nicotine, heavy metals, and multiple carcinogenic substances, many of which possess neurotoxic properties and may disrupt normal central nervous system development in fetuses and children^10^. Animal studies and neuroimaging investigations have demonstrated that early-life SHS exposure can affect the dopaminergic system and modify cortical brain structures, thereby impairing attention regulation and impulse control^11,12^. SHS exposure during pregnancy and childhood represent distinct biological exposure windows that may have different implications for prevention and intervention strategies. Epidemiological investigations have consistently demonstrated that maternal smoking during pregnancy is associated with an increased risk of ADHD in offspring^8,13^. However, evidence regarding the relationship between postpartum household SHS exposure and ADHD remains inconsistent and comparatively limited. Several studies have reported a significant association between SHS exposure and ADHD symptoms in children, whereas other investigations have indicated that the association weakens or disappears after adjustment for confounding variables, including demographic characteristics and socioeconomic status^9,14,15^. These inconsistencies may reflect differences in sample size, exposure assessment approaches, and insufficient adjustment for potential confounders. Furthermore, previous investigations have primarily concentrated on specific populations or relatively small cohorts, highlighting the need for large-scale analyses using nationally representative datasets.

To address these limitations, the objective of this study was to systematically evaluate the association between household SHS exposure and ADHD risk among US children.

## METHODS

### Data sources and study population

Data for the present secondary analysis were obtained from three consecutive NHANES cycles (1999–2000, 2001–2002, and 2003–2004), because these survey cycles were the only datasets containing complete physician-diagnosed ADHD information. NHANES is a cross-sectional survey designed to evaluate the health and nutritional status of the US population through a complex, stratified, multistage probability sampling design. The survey protocol was approved by the National Center for Health Statistics Research Ethics Review Board, and written informed consent was obtained from all participants.

**Figure 1:**
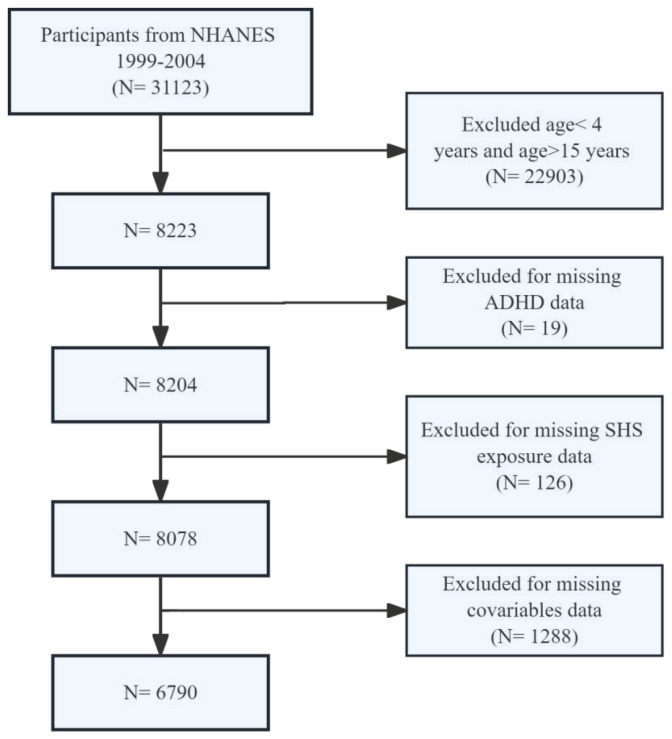
Flowchart of participant selection

The initial sample included 31126 participants. ADHD-related information was collected for individuals aged 4–19 years, whereas the early childhood questionnaire component targeted participants from birth to 15 years of age. Therefore, the study population was restricted to children aged 4–15 years. Participants younger than 4 years or older than 15 years were excluded (n=22903). Additional exclusions included participants with missing ADHD information (n=19), incomplete household SHS exposure data (n=126), or missing covariate information (n=1288). Consequently, the final analytical sample consisted of 6790 children. A detailed participant selection flowchart is presented in Figure 1.

### Definitions of variables

*Exposure variable*

Household SHS exposure was assessed using questionnaire item SMD410: ‘Does anyone who lives here smoke cigarettes, cigars, or pipes anywhere inside this home?’. Participants responding ‘Yes’ were categorized into the exposed group, whereas those responding ‘No’ were classified into the non-exposed group^16^.

*Outcome variable*

ADHD diagnosis was determined based on parental reports of physician-diagnosed ADHD. ADHD status was evaluated using questionnaire item MCQ060: ‘Has a doctor or health professional ever told [you/SP] that [you/(s)he/SP] had attention deficit disorder?’. Participants responding ‘Yes’ were classified in the ADHD group, whereas those responding ‘No’ were assigned to the control group^17^.

### Covariates

Based on previous literature and clinical relevance, the following covariates were included in the analysis: age, sex, race, family poverty-income ratio (PIR), body mass index (BMI), maternal age at childbirth, birth weight, smoking during pregnancy, asthma, and health insurance status^13,18-20^. Age was categorized into four groups: 4–6, 7–9, 10–12, and 13–15 years^13^. Sex was categorized as male or female. Race was classified as Mexican American, non-Hispanic White, non-Hispanic Black, or other race^19^. PIR was categorized into three groups based on previous studies: ≤1.3, 1.3–3.5, and >3.5^20^. BMI was calculated as body weight in kilograms divided by height in meters squared. According to BMI growth charts published by the US Centers for Disease Control, children were categorized as underweight (<5th percentile), normal weight (5th–85th percentile), overweight (85th–95th percentile), or obese (≥95th percentile)^18^. Birth weight values were converted into grams for consistency and categorized as: <2500, 2500–4000, or >4000 g^18^. Smoking during pregnancy was defined according to responses to questionnaire item ECQ020: ‘Did (SP name’s) biological mother smoke at any time while she was pregnant with (him/her)?’^18^. Asthma status was determined according to self-reported physician diagnosis using questionnaire item MCQ010: ‘Has a doctor or other health professional ever told (you/ SP) that (you have/(s)he/SP has) asthma?’^18^. Health insurance coverage was assessed using questionnaire item HID010: ‘Are you/is SP covered by health insurance or some other kind of healthcare plan?’^18^.

### Statistical analysis

All analyses incorporated the complex sampling framework of NHANES and applied weighted statistical procedures to ensure national representativeness and analytical robustness. NHANES utilizes a multistage probability sampling design to achieve representativeness of the US population. In accordance with National Center for Health Statistics recommendations, sampling weights (WTMEC4YR/WTMEC2YR), strata (SDMVSTRA), and primary sampling units (SDMVPSU) were incorporated into all analyses to account for the complex survey design. Specifically, the sampling weight variable ‘WTMEC4YR’ was applied for the 1999–2002 survey cycles, whereas ‘WTMEC2YR’ was used for the 2003–2004 cycle. Sampling weights were calculated as follows: for the 1999–2002 cycles, weights were computed as 2/3 × WTMEC4YR, whereas for the 2003–2004 cycle, weights were calculated as 1/3 × WTMEC2YR. All continuous variables demonstrated non-normal distributions according to the Shapiro–Wilk test and from a visual inspection of the histograms. Therefore, continuous variables were summarized as medians with interquartile ranges (IQR) and compared using weighted Wilcoxon rank-sum tests. Categorical variables were expressed as frequencies with weighted percentages, and comparisons between groups were performed using weighted chi-squared tests. Weighted multivariate logistic regression models were employed to evaluate the association between household SHS exposure and ADHD. Three regression models were established. Model 1 included no covariate adjustment. Model 2 adjusted for basic sociodemographic characteristics, including age, sex, race, and PIR. Model 3 further adjusted for BMI, maternal age at childbirth, birth weight, smoking during pregnancy, asthma, and health insurance status based on Model 2. Results were reported as odds ratios (ORs) with 95% confidence intervals (CIs). Subgroup analyses were stratified according to sex, age, race, PIR, BMI, and birth weight. To enhance the reliability and stability of the findings, sensitivity analyses were also conducted. First, multiple imputation was performed for covariates with missing values, including PIR, BMI, maternal age at childbirth, birth weight, smoking during pregnancy, asthma, and health insurance status, with the *mice* package in R employed to reduce potential bias associated with missing data. The imputed datasets were subsequently used in weighted multivariate logistic regression analyses to reassess the association between household SHS exposure and ADHD. Subsequently, weighted multivariate logistic regression analyses were repeated using serum cotinine concentrations as a surrogate biomarker of household SHS exposure. All statistical analyses were conducted using R software (version 4.5.0). A two-sided p<0.05 was considered statistically significant.

## RESULTS

### Baseline characteristics

A total of 6790 children were included in the final analysis, among whom 452 children were classified into the ADHD group, corresponding to a weighted prevalence of approximately 7.97%. As shown in Table 1, the median age of children in the ADHD group was significantly higher than that of the non-ADHD group (12.00 vs 9.00 years, p<0.001), and the proportion of male participants was also significantly greater (75.91% vs 48.35%, p<0.001). The prevalence of household SHS exposure was significantly higher among children with ADHD (36.25% vs 22.77%, p<0.001). Moreover, mothers of children in the ADHD group reported substantially higher rates of smoking during pregnancy compared with mothers in the non-ADHD group (30.18% vs 17.81%, p<0.001). In addition, children with ADHD demonstrated a significantly higher prevalence of asthma (23.87% vs 14.60%, p<0.001). Regarding demographic and health-related characteristics, children with ADHD had significantly lower maternal age at childbirth, but exhibited higher BMI values and greater rates of health insurance coverage compared with the non-ADHD group (all p<0.05).

**Table 1:** Baseline characteristics of the participants based on ADHD status in US children aged 4–15 years, NHANES 1999–2004 (weighted)

*Characteristics*	*All* *(N=6790)*	*Control* *(N=6338)*	*ADHD* *(N=452)*	*p*
**Age** (years)	10.00 (7.00–13.00)	9.00 (6.00–12.00)	12.00 (9.00–13.00)	<0.001
**Gender**				<0.001
Male	3305 (50.55)	2968 (48.35)	337 (75.91)	
Female	3485 (49.45)	3370 (51.65)	115 (24.09)	
**Race**				0.002
Mexican American	2224 (11.81)	2146 (12.39)	78 (5.13)	
Non-Hispanic White	1876 (61.58)	1696 (60.89)	180 (69.64)	
Non-Hispanic Black	2113 (14.07)	1961 (14.20)	152 (12.51)	
Other Race	577 (12.54)	535 (12.52)	42 (12.72)	
**PIR**	2.12 (1.06–3.75)	2.12 (1.06–3.80)	1.83 (0.96–3.51)	0.152
**BMI** (kg/m^2^)	18.07 (15.91–21.51)	18.00 (15.87–21.47)	18.69 (16.37–21.91)	0.048
**Maternal age at childbirth** (years)	26.00 (22.00–31.00)	27.00 (22.00–31.00)	24.00 (21.00–29.00)	<0.001
**Birth weight** (g)				0.659
<2500	837 (11.24)	775 (11.10)	62 (12.83)	
2500–4000	5376 (79.97)	5030 (80.13)	346 (78.15)	
>4000	577 (8.79)	533 (8.77)	44 (9.02)	
**Smoking during pregnancy**				<0.001
No	5821 (81.20)	5487 (82.19)	334 (69.82)	
Yes	969 (18.80)	851 (17.81)	118 (30.18)	
**Asthma**				<0.001
No	5710 (84.66)	5370 (85.40)	340 (76.13)	
Yes	1080 (15.34)	968 (14.60)	112 (23.87)	
**Household SHS exposure**				<0.001
No	5302 (76.15)	4998 (77.23)	304 (63.75)	
Yes	1488 (23.85)	1340 (22.77)	148 (36.25)	
**Health insurance**				0.011
No	1090 (12.21)	1049 (12.59)	41 (7.84)	
Yes	5700 (87.79)	5289 (87.41)	411 (92.16)	

ADHD: attention deficit hyperactivity disorder. PIR: poverty income ratio. BMI: body mass index. Median (IQR) for continuous variables, p-values were calculated by weighted Wilcoxon rank-sum tests. Number (%) for categorical variables, p-values were calculated by the weighted chi-squared tests.

### Association between household SHS exposure and ADHD

Weighted multivariate logistic regression analyses demonstrated that household SHS exposure was significantly associated with ADHD risk in children (Table 2). In the unadjusted model (Model 1), children exposed to household SHS exhibited 1.928-fold higher odds of ADHD compared with non-exposed children (OR=1.928; 95% CI: 1.497–2.483; p<0.001). After adjusting for age, sex, race, and PIR in Model 2, the association remained statistically significant (AOR=1.708; 95% CI: 1.242–2.350; p=0.002). Following further adjustment for BMI, maternal age at childbirth, birth weight, smoking during pregnancy, asthma, and health insurance status in Model 3, the positive association persisted, although the magnitude of the association was slightly attenuated (AOR=1.583; 95% CI: 1.166–2.150; p=0.005).

**Table 2:** The association between SHS exposure in the household and ADHD in US children aged 4–15 years, NHANES 1999–2004 (weighted multivariate logistic regression analyses)

*Variable*	*Model 1*		*Model 2*		*Model 3*	
	*OR (95% CI)*	*p*	*AOR (95% CI)*	*p*	*AOR (95% CI)*	*p*
Household SHS exposure	1.928 (1.497–2.483)	<0.001	1.708 (1.242–2.350)	0.002	1.583 (1.166–2.150)	0.005

Model 1: no covariates were adjusted. AOR: adjusted odds ratio. Model 2: adjusted for age, gender, race and PIR. Model 3: adjusted as for Model 2 plus BMI, maternal age at childbirth, birth weight, smoking during pregnancy, asthma, and health insurance.

### Subgroup analyses and sensitivity analyses

**Figure 2:**
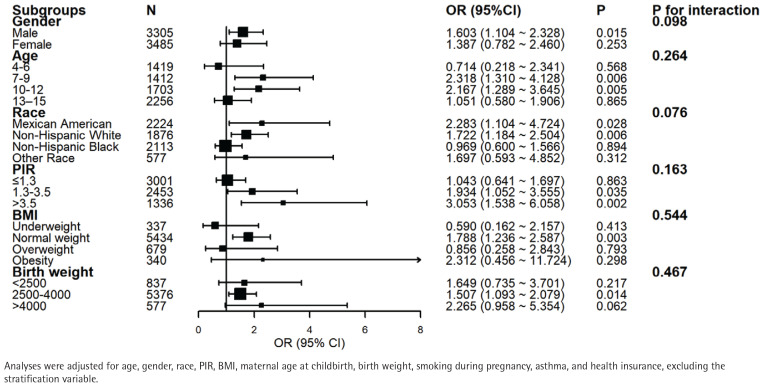
Subgroup analysis of the relationship between SHS exposure in the household and ADHD in US children aged 4–15 years, NHANES 1999–2002 (weighted multivariate logistic regression analyses)

Stratified analyses were performed to investigate the association between household SHS exposure and ADHD across subgroups defined by major demographic and clinical characteristics (Figure 2). The point estimates appeared comparatively higher in several subgroups, including males (AOR=1.603; 95% CI: 1.104–2.328; p=0.015), children aged 7–9 years (AOR=2.318; 95% CI: 1.310–4.128; p=0.006) and 10–12 years (AOR=2.167; 95% CI: 1.289–3.645; p=0.005), non-Hispanic White participants (AOR=1.722; 95% CI: 1.184–2.504; p=0.006), participants with PIR>3.5 (AOR=3.053; 95% CI: 1.538–6.058; p=0.002), children with normal BMI (AOR=1.788; 95% CI: 1.236–2.587; p=0.003), and those with normal birth weight (2500–4000 g) (AOR=1.507; 95% CI: 1.093–2.079; p=0.014). Nevertheless, interaction analyses across all subgroup categories did not reveal any statistically significant interaction effects (all for interactions p>0.05).

Sensitivity analyses further supported the robustness of the observed association between household SHS exposure and ADHD. First, baseline characteristics were compared between participants excluded because of missing covariate information (n=1228) and those included in the final analysis (n=6790) (Supplementary file Table 1). The two groups differed significantly with respect to racial composition (p<0.05), whereas no statistically significant differences were identified for age, sex, household SHS exposure, or ADHD prevalence (all p>0.05). Following multiple imputation for missing covariate data and adjustment for potential confounding variables, the association between household SHS exposure and ADHD remained statistically robust (AOR=1.438; 95% CI: 1.077–1.920; p=0.020) (Supplementary file Table 2). Second, serum cotinine concentrations were used as a surrogate biomarker of household SHS exposure. After excluding 971 participants with missing serum cotinine data, 5819 children remained eligible for analysis. Median serum cotinine concentrations were significantly higher among children with ADHD (median [IQR]: 0.24 [0.04–1.25] vs 0.08 [0.03–0.52] ng/mL, p<0.001). Furthermore, after logarithmic transformation of serum cotinine concentrations and adjustment for confounding variables, a strong positive association was observed between log-transformed cotinine levels and ADHD (AOR=1.762; 95% CI: 1.181–2.628; p=0.007) (Supplementary file Table 3).

## DISCUSSION

The present study systematically evaluated the association between household SHS exposure and ADHD among children using nationally representative NHANES data. The findings demonstrated that household SHS exposure was significantly associated with ADHD risk, even after adjustment for multiple demographic, socioeconomic, perinatal exposure, and comorbidity-related variables, with approximately 65% higher odds relative to non-exposed children. These findings suggest that household SHS exposure may represent an important environmental factor associated with ADHD among US children and provide additional epidemiological evidence regarding the environmental etiology of ADHD.

The findings of the present study are generally consistent with most previous investigations. The weighted prevalence of ADHD among US children in the present sample was 7.97%, which closely corresponds to the estimated prevalence of childhood ADHD reported in the 2003 National Survey of Children’s Health dataset (7.8%)^21^. The present analysis additionally confirmed a significant association between household SHS exposure and ADHD among children. A previous meta-analysis demonstrated that postnatal SHS exposure increased ADHD risk in children by approximately 60% (OR=1.60; 95% CI: 1.37–1.87), which is highly comparable to the OR of 1.583 observed in Model 3 of the current study^9^. Another small-scale investigation reported that 88.6% of children with ADHD were exposed to household SHS, compared with 46.7% of non-ADHD participants^22^. Furthermore, a cross-sectional study conducted in China demonstrated that environmental smoke exposure was associated with increased ADHD symptoms and impaired executive function among young adults^23^. Collectively, these converging findings indicate that household SHS exposure may represent a relevant environmental factor associated with ADHD. By utilizing nationally representative NHANES data, the present study further strengthens the existing evidence base.

Several biological mechanisms may explain the significant association between household SHS exposure and ADHD among children. First, nicotine, the major active component of SHS, can cross the blood–brain barrier and influence the development of the cerebral cortex and limbic system^24^. Second, SHS exposure may impair attentional regulation and behavioral inhibition through alterations in the sensitivity and regulatory function of dopaminergic and noradrenergic neurotransmitter systems^25^. Third, SHS contains substantial quantities of free radicals and harmful particulate matter capable of activating oxidative stress pathways^26^. These processes may promote neuroinflammation and subsequently influence cognitive and behavioral development^27^. Fourth, gene–environment interactions may further exacerbate phenotypic manifestations of ADHD^28^. Previous studies have suggested that polymorphisms in dopamine receptor-related genes may be associated with smoking behavior, thereby increasing the likelihood of childhood SHS exposure^29^.

The present study identified a positive association between household SHS exposure and childhood ADHD. Nevertheless, an important limitation that warrants consideration is the potential influence of unmeasured genetic confounding. ADHD is among the most highly heritable psychiatric disorders, with twin studies estimating heritability ranging from 74% to 88%^30^. Importantly, accumulating evidence indicates that young adults with ADHD are approximately two to three times more likely to smoke than individuals without ADHD^31^. This finding suggests a plausible confounding pathway: parents with underlying ADHD or executive function impairments may be more likely to smoke inside the household, and these same parents may also transmit their genetic susceptibility for ADHD to their offspring. Therefore, the observed association between household SHS exposure and childhood ADHD may be partially attributable to shared genetic liability rather than a direct environmental causal effect. Because the present study employed a cross-sectional design with self-reported exposure measures, it cannot disentangle inherited genetic susceptibility from environmental causation. Future investigations using sibling-comparison or genetically informed study designs are necessary to determine whether the association persists after controlling for unmeasured genetic confounding.

However, several discrepancies exist between the present findings and previous studies. Zhang et al.^15^ reported no significant association between early childhood SHS exposure (1–3 years of age) and ADHD among Chinese preschool children. These inconsistencies may result from differences in sample composition, population characteristics, subgroup definitions, and analytical methodologies across studies. The present investigation included a relatively large sample of 6790 children derived from the NHANES database, thereby providing a more comprehensive and nationally representative assessment of the US pediatric population. In contrast, smaller sample sizes in some earlier studies may have reduced statistical power to detect significant associations after adjustment for multiple confounding variables.

The present findings additionally extend previous research, which has primarily concentrated on heterogeneity according to sex and age while insufficiently examining factors such as race, PIR, BMI, and other demographic indicators. Although the present results suggested that the positive association between household SHS exposure and ADHD appeared slightly stronger among children aged 7–12 years, the interaction effect did not achieve statistical significance (interaction p>0.05). This observation may be related to critical developmental periods of the prefrontal cortex during this age range, as well as increased time spent within the household environment^32^. By contrast, the association was not statistically significant among adolescents aged 13–15 years. This finding may reflect progressive maturation of brain development during adolescence, increased tolerance to low-to-moderate tobacco SHS exposure, greater time spent outside the home, or potential confounding effects of active smoking behaviors emerging during adolescence^33,34^. The observed association appeared stronger among male children, which is consistent with the known sex distribution of ADHD. ADHD prevalence is generally higher among males than females, potentially reflecting increased susceptibility of the male nervous system to nicotine exposure or sex-related hormonal differences influencing neurotransmitter regulation^35^. Notably, the strongest association was identified among children from households with PIR>3.5, which differs from the conventional perspective that lower socioeconomic status constitutes a risk factor for childhood ADHD^36^. One possible explanation for this is that children from higher PIR households may have improved access to healthcare and educational resources, thereby increasing rates of ADHD diagnosis and reporting while reducing diagnostic misclassification bias. Alternatively, families with higher PIR may exhibit distinct indoor smoking patterns or possess additional unmeasured confounding factors influencing this relationship^37^. Interestingly, statistically significant associations were observed only among children with normal birth weight and normal BMI. The absence of significant findings in other subgroups may reflect insufficient sample size and consequently limited statistical power to detect weaker associations. Furthermore, children with extreme anthropometric characteristics may have underlying congenital or metabolic disorders that obscure or attenuate the independent effects of household SHS exposure. However, no statistically significant interaction effects were identified across subgroup analyses, indicating that none of these variables significantly modified the observed association. These subgroup findings should therefore be interpreted cautiously because of limited statistical power and the potential influence of multiple comparisons. Future large-scale studies are required to validate these observations.

### Strengths and limitations

Despite several strengths of the present study, including the large sample size, nationally representative dataset, and comprehensive adjustment for confounding variables, several limitations should be acknowledged. First, the cross-sectional design precludes causal inference and prevents clarification of temporal relationships. Second, ADHD status was assessed using a lifetime prevalence question, whereas household SHS exposure was measured at the time of the survey, creating a substantial temporal mismatch. Consequently, SHS exposure reported during the survey period may have occurred after ADHD diagnosis, thereby preventing interpretation of the observed association as evidence of causality and precluding exclusion of reverse causation. Third, both ADHD diagnosis and household SHS exposure were based on parental or self-reported information, which may introduce detection bias and social desirability bias. Specifically, undiagnosed or misdiagnosed ADHD cases may have been systematically overlooked, whereas smoking behaviors may have been underreported because of social stigma. Fourth, exposure dose, duration, and smoking cessation status were not assessed, limiting evaluation of potential dose-response relationships. Fifth, although the NHANES dataset is nationally representative, the NHANES 1999–2004 cycles are relatively old, which may limit the generalizability of these findings to contemporary children and to populations with different healthcare systems or sociocultural contexts. Finally, despite adjustment for multiple confounding variables, residual confounding cannot be completely excluded. Important unmeasured factors include parental mental health conditions, such as maternal depression and parental stress, as well as exposure to emerging tobacco products including electronic cigarettes^30,38^. These variables should be comprehensively addressed in future longitudinal investigations.

## CONCLUSIONS

Based on NHANES data collected from 1999–2004, the present study demonstrated that household SHS exposure was significantly associated with an increased risk of ADHD among children, and this association remained robust after adjustment for multiple potential confounding variables. Reducing household SHS exposure may contribute to lowering the risk of ADHD in children; however, longitudinal investigations are required to further validate this relationship. Future prospective cohort studies and mechanistic investigations are necessary to clarify the causal nature of this association and to elucidate the underlying biological mechanisms.

## Supplementary Material



## Data Availability

The data supporting this research are publicly available from the National Health and Nutrition Examination Survey (NHANES, http://www.cdc.gov/nchs/nhanes/).
