# Supplementary material for: Association between household secondhand smoke exposure and ADHD in US children aged 4–15 years: Evidence from NHANES 1999–2004
Source: Tob Induc Dis. 2026 Jul 16;24:10.18332/tid/222368. doi: 10.18332/tid/222368 (PMC13377825; doi:10.18332/tid/222368)
Supplement: Supplementary file 1 [file TID-24-112-s1.pdf]

Tobacco Induced Diseases

Supplementary file

© 2026 Bao Y. et al.

DOI:
10.18332/tid/222368

The content has been provided by the author(s) and has not been reviewed, verified, or endorsed by European Publishing. It may not have undergone peer review. The views, opinions, and recommendations expressed are solely those of the author(s) and do not necessarily reflect the position of European Publishing. European Publishing accepts no responsibility or liability for any consequences arising from the use of, or reliance on, this content.

Supplementary Table S1. The baseline characteristics of participants based on missing covariates data in US children aged 4–15 years: NHANES 1999-2004 (weighted)

**Characteristics**	**[ALL]** *N=8078*	**Excluded** *N=1288*	**Included** *N=6790*	***p***
Age, years	10.00 [7.00, 13.00]	9.00 [7.00, 13.00]	10.00 [7.00, 13.00]	0.641
Gender, n(%)				0.558
Male	3961 (50.73%)	656 (52.29%)	3305 (50.55%)	
Female	4117 (49.21%)	632 (47.71%)	3485 (49.45%)	
Race, n(%)				<0.001
Mexican American	2678 (12.33%)	454 (16.83%)	2224 (11.81%)	
Non-Hispanic White	2146 (59.89%)	270 (45.36%)	1876 (61.58%)	
Non-Hispanic Black	2568 (14.84%)	455 (21.52%)	2113 (14.07%)	
Other Race	686 (12.93%)	109 (16.30%)	577 (12.54%)	
Household SHS exposure, n(%)				0.050
No	6325 (76.66%)	1023 (81.03%)	5302 (76.15%)	
Yes	1753 (23.34%)	265 (18.97%)	1488 (23.85%)	
ADHD, n(%)				0.414
Control	7543 (91.89%)	1205 (90.70%)	6338 (92.03%)	
ADHD	535 (8.11%)	83 (9.30%)	452 (7.97%)	

ADHD: attention deficit hyperactivity disorder. Median [IQR] for continuous variables, *p*-values were calculated by weighted Wilcoxon rank-sum tests. Number(%) for categorical variables, *p*-values were calculated by the weighted chi-square tests.

Supplementary Table S2. The association between SHS exposure in the household and ADHD after multiple imputation of missing covariates in US children aged 4-15 years: NHANES 1999-2004 (Weighted multivariate logistic regression analyses)

	Model 1		Model 2		Model 3	
	OR (95% CI)	*p*	AOR (95% CI)	*p*	AOR (95% CI)	*p*
Household SHS exposure	1.867 (1.475, 2.362)	**<0.001**	1.625 (1.213, 2.178)	**0.003**	1.438 (1.077, 1.920)	**0.020**

Model 1: no covariates were adjusted. AOR: adjusted odds ratio. Model 2: adjusted for age, gender, race and PIR. Model 3: adjusted as for Model 2 plus BMI, maternal age at childbirth, birth weight, smoking during pregnancy, asthma, and health insurance.

Supplementary Table S3. The association between log-transformed serum cotinine levels and ADHD in US children aged 4-15 years: NHANES 1999-2004 (Weighted multivariate logistic regression analyses)

	Model 1		Model 2		Model 3	
	OR (95% CI)	*p*	AOR (95% CI)	*p*	AOR (95% CI)	*p*
N-transformed serum cotinine levels	1.976 (1.400, 2.789)	**<0.001**	1.915 (1.251, 2.930)	**0.004**	1.762 (1.181, 2.628)	**0.007**

Model 1: no covariates were adjusted. AOR: adjusted odds ratio. Model 2: adjusted for age, gender, race and PIR. Model 3: adjusted as for Model 2 plus BMI, maternal age at childbirth, birth weight, smoking during pregnancy, asthma, and health insurance.
